# On Ovariotomy; the Mode of Its Performance, and the Results Obtained at the London Surgical Home

**Published:** 1862-09

**Authors:** I. B. Brown

**Affiliations:** Senior Surgeon to the London Surgical Home


### ON OVARIOTOMY;

### THE MODE OF ITS PERFORMANCE, AND THE RESULTS OBTAINED AT THE LONDON SURGICAL HOME.

By I. B. BROWN, Esq., F. R. C. S.,
*Senior Surgeon to the London Surgical Home.*

After some preliminary observations upon the operation of ovariotomy, its now recognized character, and the mortality from it being much less than that from many other capital operations, the author alluded to the statistics of Mr. Clay, of Birmingham. He believed that, highly favorable as were the results given, there was good reason for supposing that they would become still more so when the statistics were hereafter collected, subsequently to the period to which Mr. Clay's cases extended—namely, February, 1860. The conditions rendering the operation justifiable were next dwelt upon. The difficulty of diagnosis was shown, especially in cases complicated with cancer. Much would, however, depend upon the history of the patient and her family, in arriving at a correct conclusion. Adhesions at the present time rarely prove an obstacle to the completion of the operation; they are either broken through with the hand, or divided with the hand or ecraseur. When necessary to secure any by ligature, he advised the use of silver wires instead of thread or twine, allowing them to remain within the abdomen after cutting them short and close.

The author next fully described his mode of performing ovariotomy. He advises the pedicle to be enclosed in a clamp —the ordinary carpenter's calipers—as near to the tumour as possible, and kept externally. Its advantages are, that it can be removed in from one to three days; the wound heals quickly, and convalescence ensues in two or three weeks. If the pedicle proves to be very short, and pain is complained of, the clamp is to be removed in a few hours.

The preliminary measures to be adopted previous to the operation were then described, and their importance shown as bearing on the subsequent results. Amongst others, the author advised the observation of certain atmospheric conditions, and the avoidance of any proceeding when the atmosphere was low and heavy, with an absence or deficiency of ozone, and in that condition generally which we describe as depressing. If greater attention were paid to atmospheric changes, the author thought there would be much less of gangrene, pyæmia, low fever, &c., so frequently witnessed after operations.

The after-treatment was also dwelt upon, and, finally, the analysis of the cases. Of the latter, ovariotomy has been performed nineteen times by the author in the London Surgical Home up to the present time. Of these, thirteen have been recoveries, and six deaths. The details and special particulars of the whole of these cases were given in a series of tables. The ages varied from eighteen to fifty-six. Of the successful cases, eight were under the age of thirty, and five above; whilst of the unsuccessful, one was twenty-one, and five were thirty and upwards. The duration of the disease in the successful cases was from four months to six years; six were within the first year, or ten within two years, and three over the latter period. Nine were single, and four were married, and of the latter, two only had had children. Five had undergone tapping from one to three times. In the unsuccessful cases the duration of the disease was from two to ten years; four were married, three of whom had families of from three to six children. Four of the fatal cases were tapped from one to six times. The general health was very good in five of the successful cases; in six it was but middling; in one it was shattered, and in another bad. In the unsuccessful cases four had bad health; one was in good health, and another had good health up to six weeks before the operation.

With respect to the operation, the incisions varied from three to seven inches long; in eleven it did not exceed five inches. The tumors were multilocular in eleven, and unilocular in two of the successful cases; in the unsuccessful they were multilocular in four, unilocular in one, and more or less solid in the sixth, containing hair, teeth, bones, &c., and no doubt congenital. Adhesions were found in all except four of the successful and one of the unsuccessful cases. These varied very much, being either very slight and easily broken down, or firm, strong, and unyielding; some were numerous in all directions, requiring to be cut or ligatured. Chloroform was given in all the cases; in two it had to be discontinued, but the patient suffered no pain; in two instances ether was substituted during the latter part of the operation. The pedicle was retained outside of the abdomen in all but two of the cases, the calipers being used for the purpose of holding it. In all the operations performed lately, the wound had been closed by silver-wire sutures, simply twisted. Of the causes of death in the six fatal cases, in two instances it clearly arose directly from the operation itself; in the other four, conditions were found which chiefly brought about this result; in one case—that of the solid tumor—there was much old disease found; in another, the patient had been a hard drinker, was tapped five times, the belly being filled with 45 pints of ascitic fluid, independently of the contents of the ovarian cyst, there was softening of the liver, and death occurred in six days. In the other two cases, diarrhœa carried off both—one in eight days, and the other in eighteen after the operation. In one of these, cancer of the duodenum was found, wholly unsuspected during life. The author concluded by stating that all these operations had been witnessed by gentlemen from various parts of the world, of Great Britain, and the metropolis, many of whom had watched the results from day to day and week to week.—*London Lancet.*

